# The values of *AHCY* and *CBS* promoter methylation on the diagnosis of cerebral infarction in Chinese Han population

**DOI:** 10.1186/s12920-020-00798-7

**Published:** 2020-11-02

**Authors:** Xiaodong Li, Shufang Bu, Ran Ran Pan, Cong Zhou, Kun Qu, Xiuru Ying, Jie Zhong, Jianhao Xiao, Qian Yuan, Simiao Zhang, Laura Tipton, Yunliang Wang, Youping Deng, Shiwei Duan

**Affiliations:** 1 Department of Neurology, Zhengzhou Central Hospital Affiliated to Zhengzhou University, Zhengzhou, 450006 Henan China; 2 School of Medicine, Ningbo University, Ningbo, Zhejiang, 315211 China; 3 Department of Neurology, the 960th of Hospital of PLA, Zibo, 255330 Shandong China; 4 Department of Neurology, the Second Affiliated Hospital, Zhengzhou University, Zhengzhou, 450014 Henan China; 5 Bioinformatics Core, Department of Complementary and Integrative Medicine and John A. Burns School of Medicine, University of Hawai’i, Honolulu, HI 96822 USA

**Keywords:** Cerebral infarction, DNA methylation, *AHCY*, *CBS*, qMSP

## Abstract

**Background:**

The goal of our study is to investigate whether the methylation levels of AHCY and CBS promoters are related to the risk of cerebral infarction by detecting the methylation level of AHCY and CBS genes.

**Methods:**

We extracted peripheral venous blood from 152 patients with cerebral infarction and 152 gender- and age-matched healthy controls, and determined methylation levels of AHCY and CBS promoters using quantitative methylation-specific polymerase chain reaction. We used the percentage of methylation reference (PMR) to indicate gene methylation level.

**Results:**

We compared the promoter methylation levels of two genes (AHCY and CBS) in peripheral blood DNA between the cerebral infarction case group and the control group. Our study showed no significant difference in AHCY promoter methylation between case and control. Subgroup analysis by gender showed that the methylation level of AHCY in males in the case group was lower than that in the control group, but the difference was not statistically significant in females. In a subgroup analysis by age, there was no significant difference in the AHCY methylation level between the case and control in the young group (≤44 years old). However, the level of AHCY gene methylation in the middle-aged group (45–59 years old) was significantly higher and the aged group (≥60 years old) was significantly lower than that in the control groups. However, CBS promoter methylation levels were significantly lower in the case group than in the control group (median PMR: 70.20% vs 104.10%, *P* = 3.71E-10). In addition, the CBS methylation levels of males and females in the case group were significantly lower than those in the control group (male: 64.33% vs 105%, *P* = 2.667E-08; female: 78.05% vs 102.8%, *P* = 0.003). We also found that the CBS levels in the young (23–44), middle-aged (45–59), and older (60–90) groups were significantly lower than those in the control group (young group: 69.97% vs 114.71%; *P* = 0.015; middle-aged group: 56.04% vs 91.71%; *P* = 6.744E-06; older group: 81.6% vs 119.35%; *P* = 2.644E-04). Our ROC curve analysis of CBS hypomethylation showed an area under the curve of 0.713, a sensitivity of 67.4%, and a specificity of 74.0%.

**Conclusion:**

Our study suggests that hypomethylation of the CBS promoter may be closely related to the risk of cerebral infarction and may be used as a non-invasive diagnostic biomarker for cerebral infarction.

## Background

Stroke is one of the leading causes of death and disability [1], with ischemic stroke (cerebral infarction) accounting for 60 to 80% of all strokes [2]. Cerebral infarction is a complex disease that is affected by both environmental risk factors and genetic factors [3, 4]. Common risk factors for cerebral infarction include age, hypertension, diabetes, hyperlipidemia, smoking, and atrial fibrillation, etc. [5]. DNA methylation is an epigenetic modification that links the roles between heredity and the environment [6]. The mechanism depends on the catalysis of DNA methyltransferase (DNMT), which in turn causes the transfer of methyl groups from s-adenosylmethionine (SAM) to specific segments in the DNA sequence, thereby changing the function of the genome [7]. Therefore, the level of SAM is closely related to DNA methylation. Xiao et al. [8] showed that PON1 and PON3 methylation were associated with the risk of male cerebral infarction. There are several DNA methylation studies related to stroke [9]. High homocysteine (Hcy) leads to hypermethylation of the TM gene and further induction of TM gene silencing, which may play an important role in the development and progression of ischemic brain injury [10]. DNA methylation of CDKN2B may play a potential role in arterial calcification in patients with cerebral infarction [11]. People with a lower degree of LINE-1 methylation have a higher risk of stroke [12]. DNA methylation inhibitor Zebularine confers neuroprotective effects on the ischemic rat brain and further supports the hypothesis that DNA methyltransferase promotes delayed ischemic brain injury [13].

In the normal fasting state, the human plasma Hcy concentration is 5–15 μmol. Hcy, as a sulfur-containing amino acid in the human body, can be summarized as: (1) further metabolized to cystathionine and cysteine; (2) re-converted to methionine; (3) as a -Based receptors participate in choline metabolism; (4) participate in the folic acid cycle as an essential substrate. Wald et al. have shown that a 5 μmol/L increase in plasma Hcy leads to a 59% increased risk of cerebral infarction; a decrease in Hcy of 3 μmol/L reduces the risk of cerebral infarction by 24% [14]. In 2011, the American Heart Association and the Association for Cerebral Infarction jointly issued a primary prevention guideline for cerebral infarction, which increases the risk of ischemic cerebral infarction by two to three times as plasma Hcy levels increase [15]. In addition, a meta-analysis of a prospective cohort study found that after a mean follow-up of 7.3 years, serum HHcy was reduced by 3 μmol/L, and stroke risk was reduced by 24% [16]. Ashjazadeh et al. found high serum Hcy associated with stroke in the Iranian population [17]. Hankey et al. found elevated serum Hcy in stroke patients in Australia as an independent risk factor for ischemic stroke [18]. Hyperhomocysteinemia (HHcy) is commonly recognized as an independent risk factor for stroke [19, 20].

**Fig. 1:**
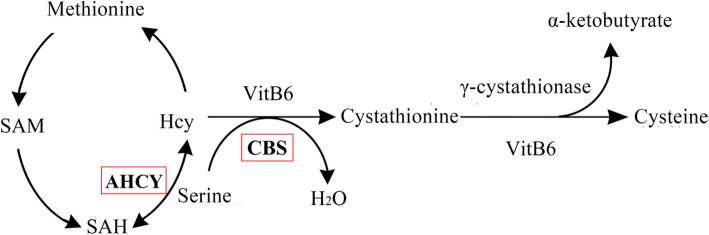
Hcy metabolic pathway. SAM: S-adenosylmethionine; SAH: S-adenosine homocysteine; Hcy: homocysteine; CBS: cystathionine beta synthase

AHCY encodes S-adenosine homocysteine hydrolase (SAH), whose function is to catalyze the reversible hydrolysis of SAM to produce adenosine (Ado) and Hcy (Fig. 1). SAH may control the number of methylated donors by regulating the level of intracellular SAM [21]. Plasma Hcy concentrations may increase due to genetic defects in related enzymes [22, 23]. AHCY methylation may affect the activity of the enzyme, causing a metabolic disorder of Hcy, leading to HHcy. However, the association between AHCY methylation and cerebral infarction has not previously been studied.

In addition, CBS-encoded cystathionine beta-synthase (CBS) catalyzes the sulfur metabolism of Hcy (Fig. 1). In vivo, Hcy can be irreversibly catalyzed by CBS to produce cysteine (Fig. 1). At present, many studies have explored the relationship between CBS mutations and stroke, of which T833C point mutation is the most common [24–27]. This mutation replaces threonine with isoleucine, resulting in the inactivation of CBS, blocking Hcy degradation, and ultimately leading to abnormal accumulation of Hcy (HHcy) in the body [28].

The association between AHCY and CBS promoter methylation and stroke studies has not been reported so far. In this study, we examined the levels of AHCY and CBS promoter methylation in peripheral blood from patients with cerebral infarction and healthy controls. Our goal is to assess whether promoter methylation of AHCY and CBS can be used as a diagnostic biomarker for the risk of cerebral infarction.

## Methods

### Sample collection

From October 2016 to April 2017, we selected 152 hospitalized patients with cerebral infarction from the 960th Hospital of the People’s Liberation Army. Patients were tested by brain CT or MRI, in strict accordance with the diagnostic criteria of the Fourth National Cerebrovascular Disease Conference [29]. The patients included 112 males and 40 females with an age range of 23 to 88 years and an average age of 60.37 ± 12.02 years. Then, we selected 152 healthy people as controls, including 112 males and 40 females with an age range of 30 to 90 years and an average age of 60.45 ± 12.23 years. All selected individuals excluded serious diseases, including malignant tumors, neurodegenerative diseases, peripheral vascular disease or peripheral vascular thrombosis, blood diseases, severe liver and kidney diseases, endocrine and metabolic diseases, tuberculosis, or connective tissue diseases. We extracted clinical information from participants from the medical records. We used the American Beckman AU5800 automatic biochemical analyzer and its supporting kit to detect the Hcy level of blood. Our research was approved by the hospital’s ethics committee. In addition, all subjects provided informed consent.

### DNA extraction and methylation detection

We extracted peripheral blood DNA using a DNA extraction kit (ZhiShan Biotech, Xiamen, China). We then used the EZ DNA Methylation-Gold kit (Zymo Research, USA) for bisulfite modification of peripheral blood DNA [30]. Among them, methylated cytosine remains intact, and all unmethylated cytosines are converted to uracil. Uracil will be expressed as thymine in the subsequent qMSP reaction.

**Fig. 2:**
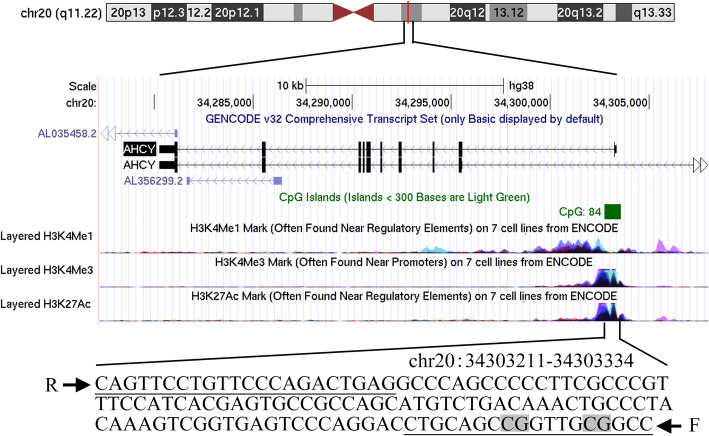
The characteristics of *AHCY* fragment in the qMSP assay. The qMSP primers were underlined and two CpG sites were in grey. F: forward primer; R: reverse primer

**Fig. 3:**
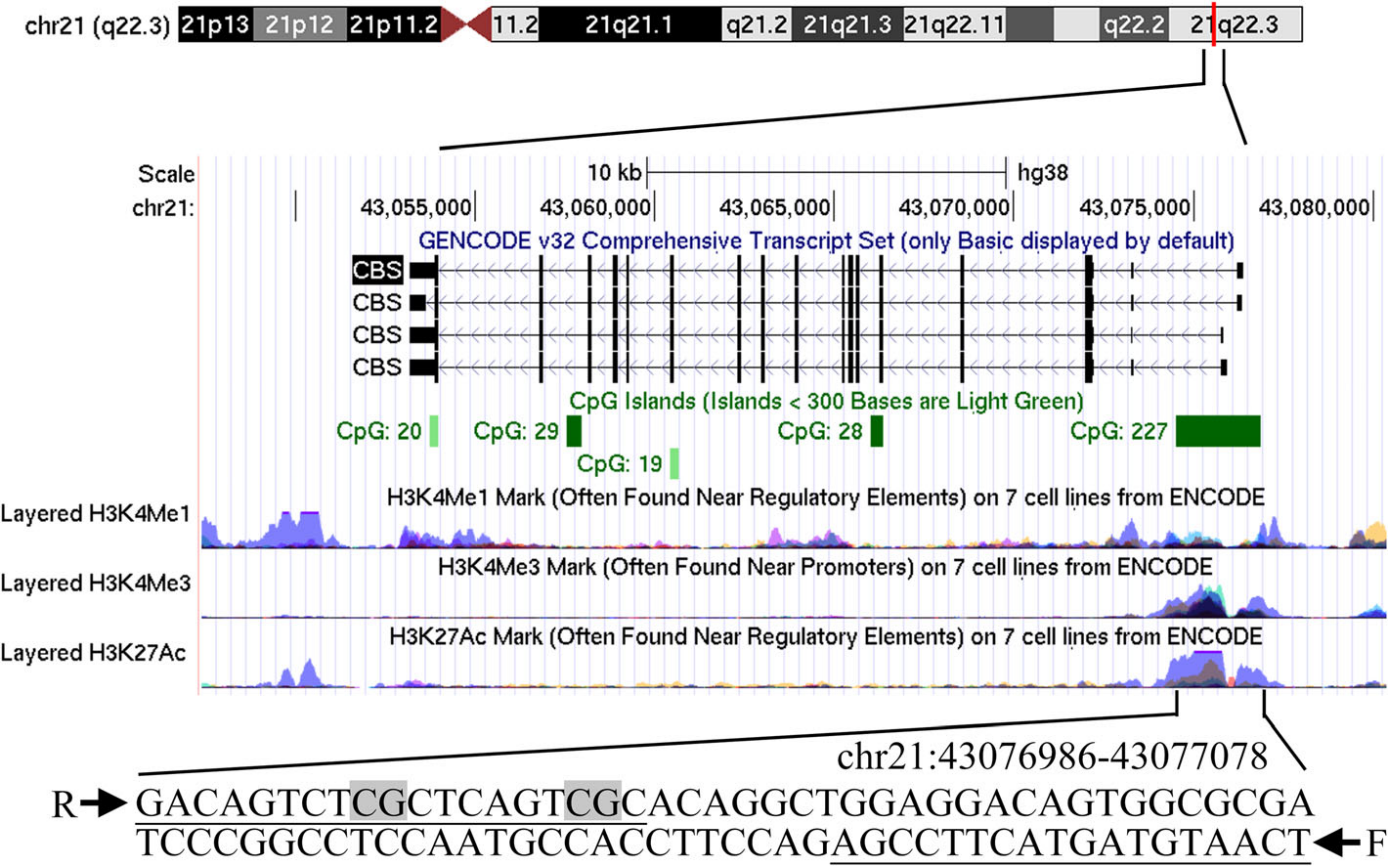
The characteristics of *CBS* fragment in the qMSP assay. The qMSP primers were underlined and two CpG sites were in grey. F: forward primer; R: reverse primer

The fragments we detected were located on the CpG island (CGI) of the promoter regions of the two genes, respectively (Fig. 2, AHCY, chr20:34303211–34,303,334; Fig. 3, CBS, chr21:43076986–43,077,078). The relevant qMSP primer sequences are listed in Table 1. The mixture components of the relevant reagents of qMSP are as previously described [31]. qMSP was performed using a 384-well transparent plate in a LightCycler 480 (Roche, Basel, Switzerland). Our qMSP chose ACTB as an internal reference to correct for differences in the loading samples. We used fully methylated human sperm DNA as a positive control and ddH2O as a blank control. Choose a 384-well plate for loading, add the sealing film after adding the sample, centrifuge in a centrifuge to remove air bubbles in the 384-well plate, and then place the 384-well plate in Roche 480 quantitative PCR instrument for measurement. The specific procedures of PCR are as follows: ① Denaturation: 95 °C, 10 min; ② Cycle (45×): 95 °C, 20 s; Annealing temperature, 20 s; 72 °C, 30 s; ③ Melting curve: 95 °C, 15 s; 60 °C, 15 s; 95 °C, continuous; ④ cooling: 40 °C, 10 min; Acquisition 1.5/°C. After the above reaction is completed, the PCR product is placed in a BiOptic Qseq100 automatic nucleic acid analyzer for analysis of the results, and the DNA methylation detection result obtained is expressed by a methylation percentage parameter (PMR). The PMR value of gene was calculated by this equation [PMR_*gene*_ = 2^-△△Ct^ × 100%, △△Ct = sample DNA (Ct _*gene*_– Ct _*ACTB*_) - fully methylated DNA (Ct_*gene*_ – Ct _*ACTB*_)]. [PMR _*CBS*_ = 2^-△△Ct^ × 100%, △△Ct = sample DNA (Ct_*gene*_ – Ct _*ACTB*_) - fully methylated DNA (Ct_*gene*_ – Ct _*ACTB*_)].

**Table 1:** Primer sequences of the qMSP assays

Gene	Primer sequence(5′- 3′)	Fragment size	Annealing temperature
*AHCY-F*	GGTCGTAATCGGTTGTAG	124 bp	58 °C
*AHCY-R*	CAATTCCTATTCCCAAACTAAA		
*CBS-F*	GGATGGAGTTATATTATGAAGGT	93 bp	56 °C
*CBS-R*	AACAATCTCGCTCAATCG		
*ACTB-F*(1)	TGGTGATGGAGGAGGTTTAGTAAGT	133 bp	58 °C
*ACTB-R*(1)	AACCAATAAAACCTACTCCTCCCTTAA		
*ACTB-F*(2)	GTGATGGAGGAGGTTTAGTAAGTT	129 bp	56 °C
*ACTB-R*(2)	CCAATAAAACCTACTCCTCCCTTAA		

**F** indicates the qMSP upstream primer; **R** indicates the qMSP downstream primer; **(1)** indicates the *ACTB* primer sequence at 58 °C, and **(2)** indicates the *ACTB* primer sequence at 56 °C

### Statistical analysis

We analyzed data using SPSS 17.0 software (SPSS Inc., Chicago, IL, USA) and GraphPad Prism 5.0 software (GraphPad Software, La Jolla, CA). We described data as median (interquartile range) or mean ± standard deviation. We used a nonparametric Mann-Whitney U test or an independent sample t-test to assess differences in DNA methylation levels between groups. We applied chi-square tests or Fisher’s exact tests to analyze the count data, and we calculated the correlation between DNA methylation levels and clinical data by Spearman rank correlation analysis. We used SPSS 17.0 software for receiver operating characteristic (ROC) curve analysis. In the ROC curve, the true positive rate (sensitivity) is plotted on the ordinate, and the false positive rate (1-specificity) is plotted on the abscissa. The best sensitivity and specificity in the ROC curve was found to show the diagnostic value of gene promoter methylation for cerebral infarction. Bilateral *P* < 0.05 was defined as statistically significant.

## Results

As shown in Table 2, our case group and control group were gender- and age-matched (*P* > 0.05). In addition, hyperlipidemia and GLU in the case group were significantly higher than those in the control group (P < 0.05). The levels of TC, HDL-C, and LDL-C in the case group were lower than those in the control group (P < 0.05). There was no significant difference in HHcy, Hcy, TG between the case group and the control group (P > 0.05).

**Table 2:** Comparison of clinical data in the case group and the control group

Factors	Case group	Control group	*P* value
Gender (Male / Female)	112/40	112/40	1^d^
Age (year)	60.37 ± 12.02	60.45 ± 12.23	0.951^a^
HHcy (Yes / No)	68/74^△^	42/62^△^	0.242 ^d^
Hyperlipemia (Yes / No)	70/81^△^	32/115^△^	**7.725E-06**^d^
Hcy (μmol/L)	1.18 ± 0.23	1.14 ± 0.16	0.068 ^c^
GLU (mmol/L)	5.70 (5.05,7.36)	5.36 (5.01,6.29)	**0.024**^b^
TC (mmol/L)	4.53 ± 1.13	5.04 ± 1.02	**5.209E-05**^a^
TG (mmol/L)	0.13 ± 0.22	0.12 ± 0.22	0.493 ^c^
HDL-C (mmol/L)	1.16 ± 0.30	1.68 ± 0.43	**5.147E-27**^a^
LDL-C (mmol/L)	2.46 ± 0.78	2.63 ± 0.67	**0.044**^a^

^a^: normal distribution, represent as ($\overline{\mathrm{x}}$ ±s); ^b^: data with non-normal distribution was represented as Q50 (Q25, Q75); ^c^: data with the log-normal distribution after conversion was represented as ($\overline{\mathrm{x}}$ ±s) said; △ indicates some samples do not have the information. Bold indicates *P* < 0.05; ^d^: Counting data

**Fig. 4:**
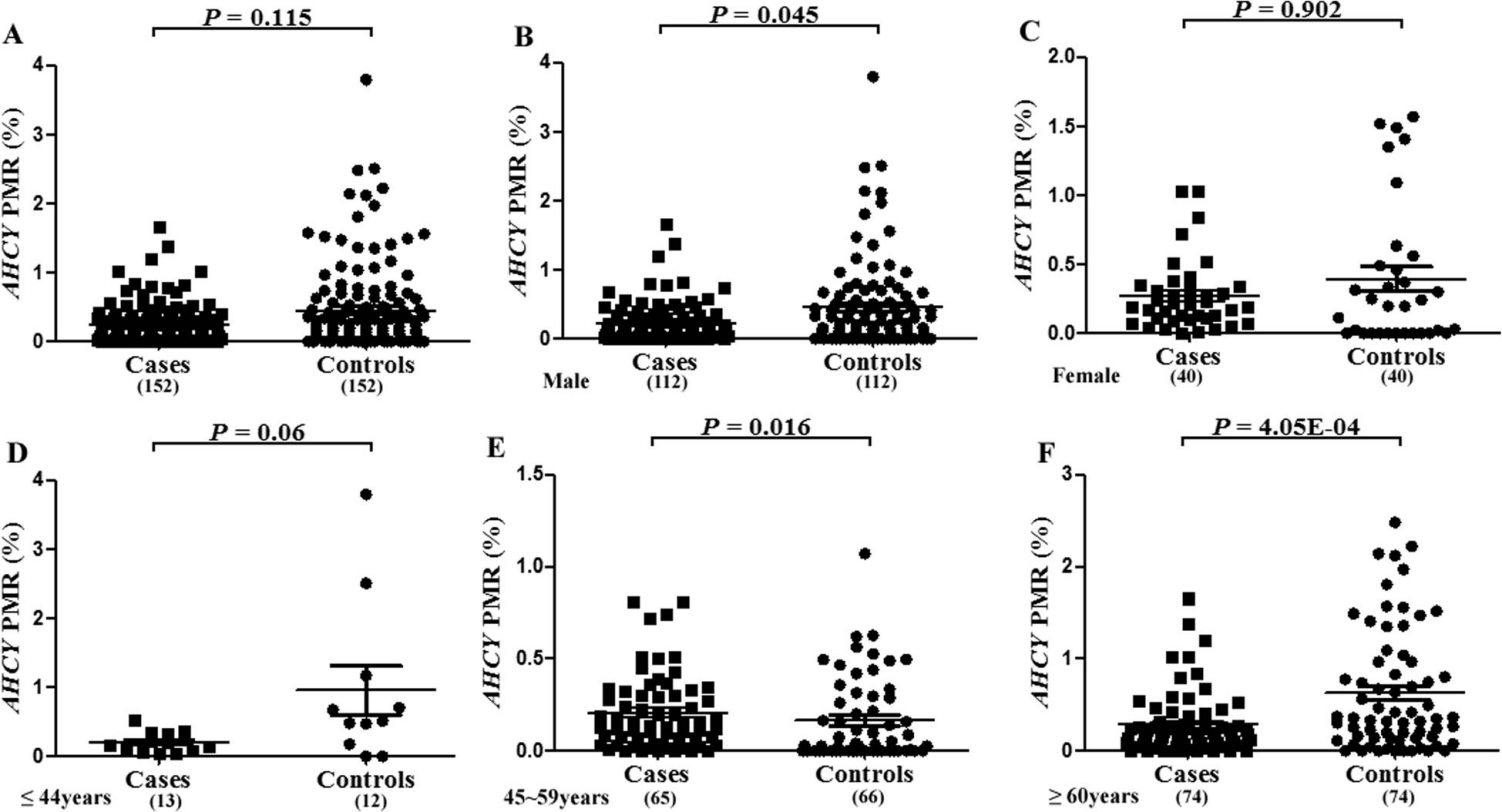
Comparison of *AHCY* methylation levels between control and case groups. **a**: Methylation level of AHCY gene in control group and case group, scatter point indicates the methylation level of experimental subjects. **b**: Methylation level of AHCY gene in males between control group and case group, scatter point indicates the methylation level of experimental subjects. **c**: Methylation level of AHCY gene in females between control group and case group, scatter point indicates the methylation level of experimental subjects. **d**: Methylation level of AHCY gene in the young group between control group and case group, scatter point indicates the methylation level of experimental subjects. **e**: Methylation level of AHCY gene in the middle-aged group between control group and case group, scatter point indicates the methylation level of experimental subjects. **f**: Methylation level of AHCY gene in the elderly group between control group and case group, scatter point indicates the methylation level of experimental subjects

**Fig. 5:**
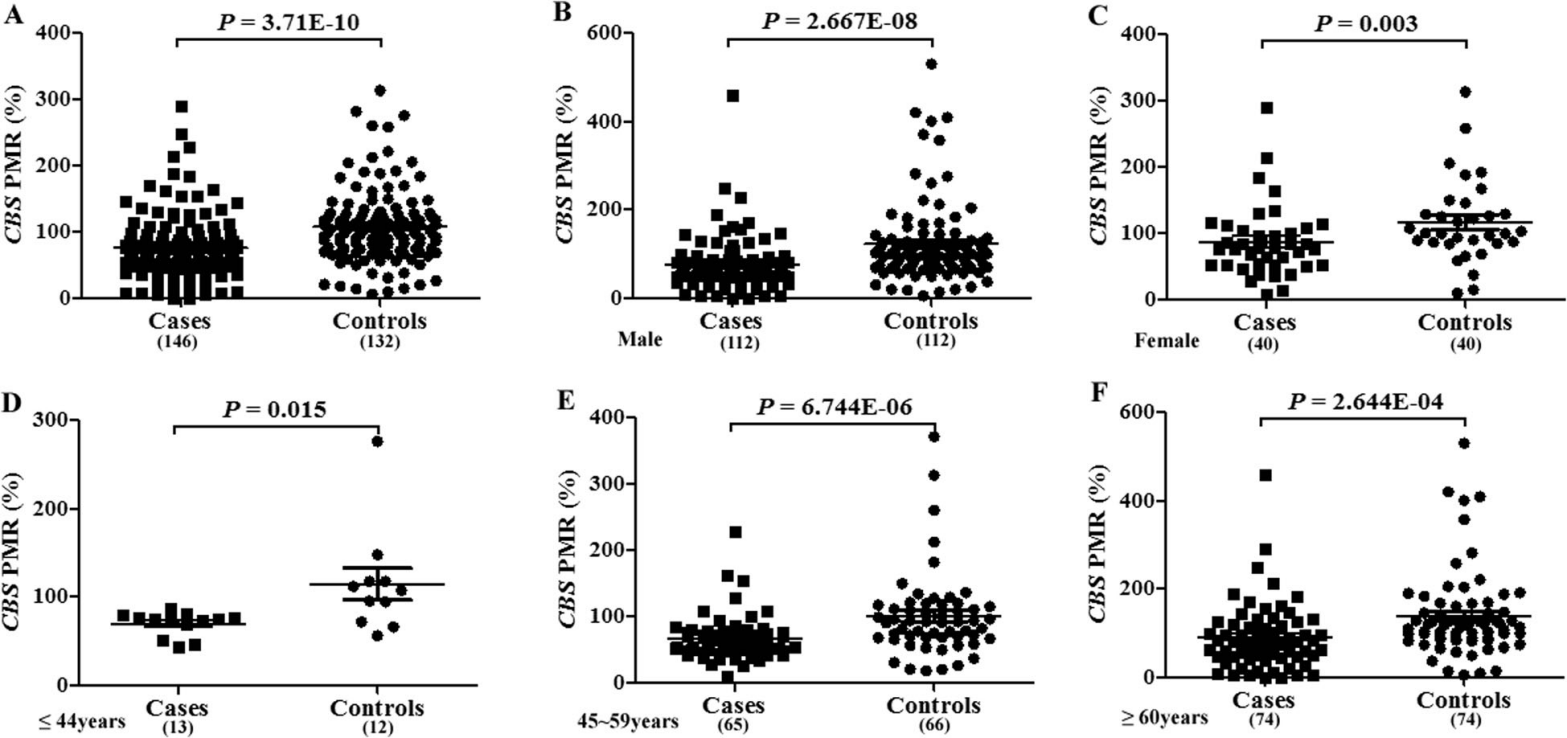
Comparison of *CBS* methylation levels between control and case groups. **a**: Methylation level of CBS gene in control group and case group, scatter point indicates the methylation level of experimental subjects. **b**: Methylation level of CBS gene in males between control group and case group, scatter point indicates the methylation level of experimental subjects. **c**: Methylation level of CBS gene in females between control group and case group, scatter point indicates the methylation level of experimental subjects. **d**: Methylation level of CBS gene in the young group between control group and case group, scatter point indicates the methylation level of experimental subjects. **e**: Methylation level of CBS gene in the middle-aged group between control group and case group, scatter point indicates the methylation level of experimental subjects. **f**: Methylation level of CBS gene in the elderly group between control group and case group, scatter point indicates the methylation level of experimental subjects

In this study, we examined the relative methylation levels of the AHCY and CBS genes in 152 patients with cerebral infarction and 152 healthy controls. We found that the PMR of the AHCY promoter region was not statistically different between the case group and the control group (*P* = 0.115, Fig. 4a). However, the CBS methylation level in the case group was significantly lower than that in the control group (median PMR: 70.20% vs. 104.10%, *P* = 3.71E-10, Fig. 5a).

Subsequently, we also conducted a subgroup analysis of gender and age stratification. In our male cases, the PMR of AHCY was lower than that of the control group (0.14 vs. 0.26, *P* = 0.045, Fig. 4b, Table 3), while the PMR of AHCY in women was not significantly different between the two groups (*P* > 0.05, Fig. 4c Table 3). Interestingly, the PMR of AHCY was higher in the middle-aged case group than in the middle-aged control group (0.14 vs. 0.03, *P* = 0.016, Fig. 4e, Table 3), while the PMR in AHCY was lower in the elderly case group than in the elderly control group (0.18 vs. 0.37, *P* = 4.05E-04, Fig. 4f, Table 3).

**Table 3:** Subgroup analysis of *AHCY* and *CBS* methylation levels by gender or age

Factors	*AHCY*^▲^ (%)	*AHCY*^△^ (%)	*P* value	*CBS*^▲^ (%)	*CBS*^△^ (%)	*P* value
Gender						
Male (*n* = 224)	0.14 (0.05,0.30)	0.26 (0.03,0.67)	**0.045**	64.33 (47.72,85.66)	105 (73.23,128.85)	2.667E-08*
Female (*n* = 80)	0.20 (0.11,0.35)	0.24 (0.00,0.86)	0.902	78.05 (51.81,107.90)	102.8 (86.11,149.90)	0.003*
Age (years)						
≤ 44 (*n* = 25)	0.20 ± 0.15	0.95 ± 1.17	0.06	69.97 ± 13.61	114.71 ± 59.62	**0.015**
45 ~ 59 (*n* = 131)	0.14 (0.04,0.31)	0.03 (0,0.31)	**0.016**	56.04 (48.07,76.23)	91.71 (68.28,118.58)	6.744E-06*
≥ 60 (*n* = 148)	0.18 (0.08,0.31)	0.37 (0.14,1.29)	**4.05E-04**	81.6 (49.37,127.45)	119.35 (87.71,167.90)	2.644E-04*

*P* value < 0.05 is in bold font. * represents *P* < 0.01. ^▲^ indicates the case group. ^△^ represents the control group

As shown in Table 4, we performed a breakdown analysis of AHCY methylation and stroke by both age and gender. We found that AHCY hypomethylation in the elderly group (age > 59) was associated with stroke risk in both men and women, whereas in middle-aged (aged between 45 and 59) women, AHCY hypermethylation was associated with stroke risk. This opposite result suggests that the risk of AHCY methylation and stroke in women needs further analysis in the future.

**Table 4:** Subgroup analysis of *AHCY* methylation levels with stoke by both gender and age

Factors	AHCY^▲^ (%)	AHCY^△^ (%)	*P* value
≤ 44 (13 vs. 12)			
Male (10 vs. 12)	0.21 ± 0.15	0.95 ± 1.17	0.065
Female (3 vs. 0)	0.15 ± 0.16	NA	NA
45 ~ 59 (65 vs. 66)			
Male (50 vs. 50)	0.19 ± 0.21	0.19 ± 0.24	0.098
Female (15 vs. 16)	0.20 (0.12,0.35)	0.00 (0.00,0.07)	**0.001**
≥ 60 (74 vs. 74)			
Male (52 vs. 50)	0.17 (0.07,0.28)	0.33 (0.11,0.83)	**0.011**
Female (22 vs. 24)	0.30 ± 0.30	0.68 ± 0.66	**0.021**

*P* value < 0.05 is in bold font. * represents *P* < 0.01. ^▲^ indicates the case group. ^△^ represents the control group. NA: not analyzed

At the same time, we found that the PMR levels of the CBS gene were significantly lower in the stratified analysis of various ages and genders (*P* < 0.01, Fig. 5, Table 3).

**Fig. 6:**
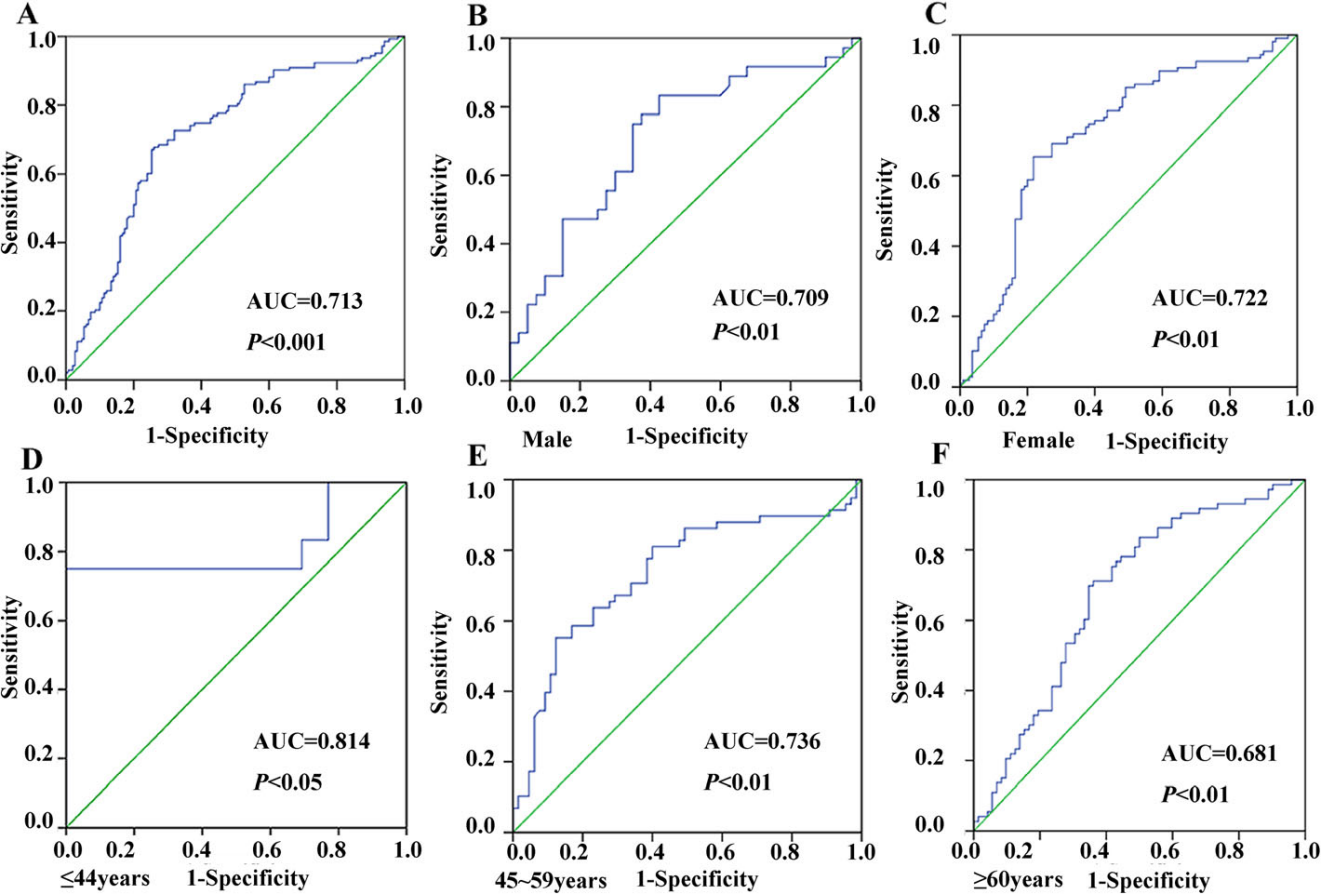
ROC curve analysis of *CBS* methylation in the cerebral infarction patients. **a**: ROC curve of methylation of the CBS gene. AUC is 0.713. **b**: ROC curve of methylation of the CBS gene in males between the control group and the case group. AUC is 0.709. **c**: ROC curve of methylation of the CBS gene in females between the control group and the case group. AUC is 0.722. **d**: ROC curve of methylation of the CBS gene in the young group between the control group and the case group. AUC is 0.814. **e**: ROC curve of methylation of the CBS gene in the middle-aged group between the control group and the case group. AUC is 0.736. **f**: ROC curve of methylation of the CBS gene in the elderly group between the control group and the case group. AUC is 0.681

Therefore, we continue to conduct a ROC curve analysis of CBS methylation. We found that the area under the ROC curve (AUC) was 0.713 (95% CI: 0.652–0.773), the sensitivity was 67.4%, and the specificity was 74.0% (Fig. 6). This indicated that CBS hypomethylation might be used as a potential biomarker for the diagnosis of cerebral infarction.

## Discussion

In this study, we first analyzed the general data between the case group and the control group and found that the two groups matched in gender and age. The prevalence of Hhcy and the level of Hcy were not different between the cerebral infarction group and the healthy control group, and it was inconsistent with the results of previous studies [32, 33]. Analysis of the reasons for no significant difference in Hhcy prevalence and Hcy level between the two groups may be related to the use of folic acid, vitamin B12, and vitamin B6 in the case group for drug intervention, insufficient sample size, and lack of sample information. The hyperlipidemia and GLU in the case group were higher than those in the control group, which was consistent with previous studies. The TC, HDL-C, and LDL-C levels in the case group were lower than those in the control group, which was considered to be related to the patients’ oral statins.

In this study, we analyzed the methylation levels of the AHCY and CBS promoters between the case and control groups. Our results indicated that hypomethylation in the CBS promoter region was significantly associated with cerebral infarction and might serve as a potential biomarker for the diagnosis of cerebral infarction. In addition, our results showed that differences in the methylation of the AHCY gene between the case group and the control group were related to gender and age. Specifically, the AHCY methylation level in male patients was lower than that in the control group. To analyze the correlation between AHCY promoter methylation and male patients with cerebral infarction. The AHCY methylation level in middle-aged patients was significantly higher than that in the control group, while the AHCY methylation level in elderly patients was lower than that in the control group. Fuso A et al. [34–36] found that the degree of bias in DNA methylation is related to many factors. When the degree of DNA methylation gradually increases, the degree of DNA methylation bias also changes, and this is mainly related to non-CpG methylation in the genome. In the current study, the correlation between the AHCY gene methylation level and ischemic stroke is inconsistent in the middle-aged and elderly groups. The bias between the middle-aged and elderly groups needs to be further explored in the future.

CBS catalyzes the irreversible synthesis of cystathionine and water from Hcy, which then produces cysteine and alpha-ketoacetate catalyzed by gamma-cystatin. We hypothesized that abnormal DNA methylation may affect enzyme activity or gene expression, which in turn leads to disorder of Hcy metabolism, but due to conditions and other limitations, it could not be further verified. To the best of our knowledge, no one has assessed the relationship between CBS methylation and cerebral infarction. Our results showed that the CBS methylation level in the case group was significantly lower than that in the control group (Fig. 5). Usually, insufficient methylation will lead to overexpression of genes, but our research shows that the CBS gene in the case group is in a state of insufficient methylation, there is no functional mechanism linking the CBS gene hypomethylation to cerebral infarction. Further research is needed to prove the effect of general methylation regulation on CBS enzyme activity and/or DNA methylation regulation on CBS gene expression impact.

Our ROC curve analysis showed CBS hypomethylation has an AUC of 0.713, a sensitivity of 67.4%, and a specificity of 74.0% (Fig. 6). Specifically, we found that 73.0% (111/152) of the 152 patients with cerebral infarction had hypomethylation of the CBS gene promoter region in contrast to only 30.0% (46/152) of the 152 healthy controls. Therefore, methylation of the CBS promoter region can be used as a potential biomarker for diagnosing cerebral infarction.

There are some limitations in this study. First, all subjects in this study were from the same region, and the sample size was not large. Therefore, the application of our results should be validated in other populations. Second, due to the limitations of the sample, we were unable to further detect CBS protein and mRNA expression. Finally, we only studied a limited area on the CGI of the two genes, and other areas of the genes for methylation need further study.

## Conclusions

In summary, our study found that hypomethylation of the CBS promoter region is a risk factor for cerebral infarction and it can serve as a potential biomarker for the diagnosis of cerebral infarction. There is an age and gender difference in the association between methylation of AHCY and cerebral infarction, and further research is needed in the future.

## Data Availability

The datasets generated in the current study are available from the corresponding author on a reasonable request.

## Abbreviations

CGI: CpG island
Hcy: homocysteine
HHcy: High homocysteine
PMR: percentage of methylation reference
qMSP: quantitative methylation-specific polymerase chain reaction
ROC: receiver operating characteristic
SAM: s-adenosylmethionine
SAH: S-adenosyl homocysteine hydrolase

## References

[1] Lloyd-Jones D, Adams R, Carnethon M, De Simone G, Ferguson TB, Flegal K, Ford E, Furie K, Go A, Greenlund K, Haase N, Hailpern S, Ho M, Howard V, Kissela B, Kittner S, Lackland D, Lisabeth L, Marelli A, McDermott M, Meigs J, Mozaffarian D, Nichol G, O’Donnell C, Roger V, Rosamond W, Sacco R, Sorlie P, Stafford R, Steinberger J, Thom T, Wasserthiel-Smoller S, Wong N, Wylie-Rosett J, Hong Y. Heart disease and stroke statistics--2009 update: a report from the American Heart Association Statistics Committee and Stroke Statistics Subcommittee. Circulation. 2009;119(3):480–6.

[2] Chinese Medical Association Neurology Branch. Chinese Medical Association Neurology Branch Cerebrovascular Disease Group. China Acute Ischemic Stroke Diagnosis and Treatment Guide 2014, Chinese. J Neurol. 2015;48:246–57.

[3] Liu Y, Ma Y, Zhang B, Wang SX, Wang XM, Yu JM (2014). Genetic polymorphisms in pre-microRNAs and risk of ischemic stroke in a Chinese population. J Mol Neurosci.

[4] Soriano-Tarraga C, Jimenez-Conde J, Giralt-Steinhauer E, Mola M, Ois A, Rodriguez-Campello A, Cuadrado-Godia E, Fernandez-Cadenas I, Carrera C, Montaner J, Elosua R, Roquer J, GeneStroke, C. The Spanish stroke genetics (2014). Global DNA methylation of ischemic stroke subtypes. PLoS One.

[5] Ankolekar S, Rewell S, Howells DW, Bath PM (2012). The influence of stroke risk factors and comorbidities on assessment of stroke therapies in humans and animals. Int J Stroke.

[6] Toporcov TN, Znaor A, Zhang ZF, Yu GP, Winn DM, Wei Q, Vilensky M, Vaughan T, Thomson P, Talamini R, Szeszenia-Dabrowska N, Sturgis EM, Smith E, Shangina O, Schwartz SM, Schantz S, Rudnai P, Richiardi L, Ramroth H, Purdue MP, Olshan AF, Eluf-Neto J, Muscat J, Moyses RA, Morgenstern H, Menezes A, McClean M, Matsuo K, Mates D, Macfarlane TV, Lissowska J, Levi F, Lazarus P, La Vecchia C, Lagiou P, Koifman S, Kjaerheim K, Kelsey K, Holcatova I, Herrero R, Healy C, Hayes RB, Franceschi S, Fernandez L, Fabianova E, Daudt AW, Curioni OA, Maso LD, Curado MP, Conway DI, Chen C, Castellsague X, Canova C, Cadoni G, Brennan P, Boccia S, Antunes JL, Ahrens W, Agudo A, Boffetta P, Hashibe M, Lee YC, Filho VW. Risk factors for head and neck cancer in young adults: a pooled analysis in the INHANCE consortium. Int J Epidemiol. 2015;44(1):169–85. 10.1093/ije/dyu255 PMC4339764 25613428

[7] Tirado-Magallanes R, Rebbani K, Lim R, Pradhan S, Benoukraf T (2017). Whole genome DNA methylation: beyond genes silencing. Oncotarget..

[8] Xiao J, Li X, Yuan Q, Zhang S, Qu K, Wu B, Wang Y, Duan S. PON1 hypermethylation and PON3 hypomethylation are associated with risk of cerebral infarction. Curr Neurovasc Res. 2019;16(2):115-22. 10.2174/1567202616666190412154407 30977447

[9] Qureshi I, Mehler M (2010). Emerging role of epigenetics in stroke: part 1: DNA methylation and chromatin modifications. Arch Neurol.

[10] Yang Z, Wang L, Zhang W, Wang X, Zhou S. Plasma homocysteine involved in methylation and expression of thrombomodulin in cerebral infarction. Biochem Biophys Res Commun. 2016;473(4):1218–22. 10.1016/j.bbrc.2016.04.042 27079234

[11] Zh S, Zhang Y, Wang L, Zhang Z, Cai B, Liu K, Zhang H, Dai M, Sun L, Xu X, Cai H, Liu X, Lu G, Xu G (2016). CDKN2B methylation is associated with carotid artery calcification in ischemic stroke patients. J Transl Med.

[12] Baccarelli A, Wright R, Bollati V, Litonjua A, Zanobetti A, Tarantini L, Sparrow D, Vokonas P, Schwartz J (2010). Ischemic heart disease and stroke in relation to blood DNA methylation. Epidemiology..

[13] Dock H, Theodorsson A, Theodorsson E (2015). DNA Methylation Inhibitor Zebularine Confers Stroke Protection in Ischemic Rats. Transl Stroke Res.

[14] Wald DS, Law M, Morris JK (2002). Homocysteine and cardiovascular disease: evidence on causality from a meta-analysis. Bmj.

[15] Goldstein LB, Bushnell CD, Adams RJ, Appel LJ, Braun LT, Chaturvedi S, Creager MA, Culebras A, Eckel RH, Hart RG, Hinchey JA, Howard VJ, Jauch EC, Levine SR, Meschia JF, Moore WS, Nixon JV, Pearson TA, C. American Heart Association Stroke, N. Council on Cardiovascular, E. Council on, Prevention, R. Council for High Blood Pressure, D. Council on Peripheral Vascular, C. Interdisciplinary Council on Quality of, R. Outcomes. Guidelines for the primary prevention of stroke: a guideline for healthcare professionals from the American Heart Association/American Stroke Association. Stroke. 2011;42(2):517–84. 10.1161/STR.0b013e3181fcb238 21127304

[16] Holmes MV, Newcombe P, Hubacek JA, Sofat R, Ricketts SL, Cooper J, Breteler MM, Bautista LE, Sharma P, Whittaker JC, Smeeth L, Fowkes FG, Algra A, Shmeleva V, Szolnoki Z, Roest M, Linnebank M, Zacho J, Nalls MA, Singleton AB, Ferrucci L, Hardy J, Worrall BB, Rich SS, Matarin M, Norman PE, Flicker L, Almeida OP, van Bockxmeer FM, Shimokata H, Khaw KT, Wareham NJ, Bobak M, Sterne JA, Smith GD, Talmud PJ, van Duijn C, Humphries SE, Price JF, Ebrahim S, Lawlor DA, Hankey GJ, Meschia JF, Sandhu MS, Hingorani AD, Casas JP. Effect modification by population dietary folate on the association between MTHFR genotype, homocysteine, and stroke risk: a meta-analysis of genetic studies and randomised trials. Lancet. 2011;378(9791):584–94. 10.1016/S0140-6736(11)60872-6 PMC3156981 21803414

[17] Ashjazadeh N, Fathi M, Shariat A (2013). Evaluation of Homocysteine level as a risk factor among patients with ischemic stroke and its subtypes. Iran J Med Sci.

[18] Hankey GJ, Eikelboom JW (2005). Homocysteine and stroke. Lancet.

[19] Clarke R, Daly L, Robinson K, Naughten E, Cahalane S, Fowler B, Graham I (1991). Hyperhomocysteinemia: an independent risk factor for vascular disease. N Engl J Med.

[20] Sawula W, Banecka-Majkutewicz Z, Kadzinski L, Jakobkiewicz-Banecka J, Wegrzyn G, Nyka W, Banecki B (2009). Homocysteine level and metabolism in ischemic stroke in the population of northern Poland. Clin Biochem.

[21] Hermes M, Osswald H, Riehle R, Piesch C, Kloor D (2008). S-Adenosylhomocysteine hydrolase overexpression in HEK-293 cells: effect on intracellular adenosine levels, cell viability, and DNA methylation. Cell Physiol Biochem.

[22] Lievers KJ, Kluijtmans LA, Blom HJ (2003). Genetics of hyperhomocysteinaemia in cardiovascular disease. Ann Clin Biochem.

[23] Gellekink H, den Heijer M, Heil SG, Blom HJ (2005). Genetic determinants of plasma total homocysteine. Semin Vasc Med.

[24] Ding R, Lin S, Chen D (2012). The association of cystathionine beta synthase (CBS) T833C polymorphism and the risk of stroke: a meta-analysis. J Neurol Sci.

[25] Fang L, Wu W, Wu YQ (2004). Relationship between polymorphisms of cystathionine beta-synthase gene and stroke. Zhongguo Wei Zhong Bing Ji Jiu Yi Xue.

[26] Dilley A, Hooper WC, El-Jamil M, Renshaw M, Wenger NK, Evatt BL (2001). Mutations in the genes regulating methylene tetrahydrofolate reductase (MTHFR C-->T677) and cystathione beta-synthase (CBS G-->A919, CBS T-->c833) are not associated with myocardial infarction in African Americans. Thromb Res.

[27] Vyletal P, Sokolova J, Cooper DN, Kraus JP, Krawczak M, Pepe G, Rickards O, Koch HG, Linnebank M, Kluijtmans LA, Blom HJ, Boers GH, Gaustadnes M, Skovby F, Wilcken B, Wilcken DE, Andria G, Sebastio G, Naughten ER, Yap S, Ohura T, Pronicka E, Laszlo A, Kozich V (2007). Diversity of cystathionine beta-synthase haplotypes bearing the most common homocystinuria mutation c.833T>C: a possible role for gene conversion. Hum Mutat.

[28] Chandra G, Pal S, Gangopadhyay PK (2006). Cystathionine beta-synthase T833C/844ins68 polymorphism and stroke. Neurol India.

[29] Chinese Medical Association Neurology Branch Cerebrovascular Disease Group Acute Ischemic Stroke Diagnosis and Treatment Guidelines Writing Group (2010). Guidelines for the diagnosis and treatment of acute ischemic stroke in China 2010. Chin J Neurol.

[30] Chen X, Yang Y, Liu J, Li B, Xu Y, Li C, Xu Q, Liu G, Chen Y, Ying J, Duan S (2017). NDRG4 hypermethylation is a potential biomarker for diagnosis and prognosis of gastric cancer in Chinese population. Oncotarget.

[31] Li B, Chen X, Jiang Y, Yang Y, Zhong J, Zhou C, Hu H, Duan S (2017). CCL2 promoter hypomethylation is associated with gout risk in Chinese Han male population. Immunol Lett.

[32] Boysen G, Brander T, Christensen H (2003). Homocysteine and risk of recurrent stroke [J]. Stroke..

[33] Omrani HQ, Shandiz EE, Qabai M (2011). Hyperhomocysteinemia, folateo and B12 vitamin in Iranian patients with acute ischemic stroke [J]. ARYA Atheroscler.

[34] Fuso A, Scarpa S, Grandoni F, Strom R, Lucarelli M (2006). A reassessment of semiquantitative analytical procedures for DNA methylation: comparison of bisulfite- and HpaII polymerase-chain-reaction-based methods. Anal Biochem.

[35] Fuso A, Ferraguti G, Scarpa S, Ferrer I, Lucarelli M (2015). Disclosing bias in bisulfite assay: "MethPrimers" underestimate high DNA methylation. PLoS One.

[36] Fuso A, Ferraguti G, Grandoni F, Ruggeri R, Scarpa S, Strom R, Lucarelli M (2010). Early demethylation of non-CpG, CpC-rich, elements in the myogenin 5′-flanking region: a priming effect on the spreading of active demethylation?. Cell Cycle.

